# Near-Complete Genome Sequencing of Influenza C Virus in the Philippines between 2014 and 2019

**DOI:** 10.1128/MRA.00900-21

**Published:** 2021-12-09

**Authors:** Daisetsu Fujita, Clyde Dapat, Emmanuel Kagning Tsinda, Mayuko Saito, Michiko Okamoto, Mariko Saito-Obata, Beatriz P. Quiambao, Socorro P. Lupisan, Hitoshi Oshitani

**Affiliations:** a Department of Virology, Tohoku University Graduate School of Medicine, Sendai, Japan; b Research Institute for Tropical Medicine (RITM), Muntinlupa, Philippines; DOE Joint Genome Institute

## Abstract

We report 19 nearly complete genome sequences of influenza C virus isolated from clinical samples recovered from children in the Philippines between 2014 and 2019.

## ANNOUNCEMENT

Influenza C virus (ICV), a member of the *Orthomyxoviridae* family, consists of an antisense single-stranded RNA genome in seven segments: polymerase basic 2 (PB2), polymerase basic 1 (PB1), polymerase 3 (P3), hemagglutinin-esterase (HE), nucleoprotein (NP), matrix (M), and nonstructural (NS). HE protein determines the antigenic properties of ICV, and currently, there are six different lineages (1). ICV mainly causes mild upper respiratory tract illnesses in children. However, sometimes it also causes lower respiratory tract illnesses, such as bronchitis, bronchiolitis, and pneumonia (2–5). ICV has been recognized as a common respiratory pathogen in humans (6–10). In our previous study, we analyzed ICV strains isolated between 2009 and 2013 in the Philippines. We determined the complete sequences of coding regions (CDS) of the HE, M, and NS gene segments and partial sequences of the four internal gene segments; we observed that the composition of the internal genes of the Philippines strains differed from that of Japanese strains (11). In this study, we aimed to determine the complete genome sequences of all seven gene segments of ICV in children in the Philippines between 2014 and 2019. The study was approved by the Ethics Committee of Tohoku University Graduate School of Medicine, Japan, and the Institutional Review Board of the RITM, Philippines.

We conducted a cohort study on Biliran Island from 2014 to 2019 and collected nasal or nasopharyngeal swabs from children with acute respiratory illness (12). We also collected samples from hospitalized children with severe pneumonia in a hospital-based study on Palawan Island from 2014 to 2016 (13). ICV was isolated and propagated in Madin–Darby canine kidney cell culture using a microplate method (11, 14), and total RNA in the cell culture supernatant was extracted using a QIAamp viral RNA minikit (Qiagen, Hilden, Germany). DNA libraries were generated using the TruSeq stranded mRNA LT sample prep kit and sequenced (100-bp paired-end reads) on a NovaSeq 6000 S4 flow cell on the Illumina NovaSeq 6000 platform at Macrogen Japan Corporation (Tokyo, Japan). CLC Genomics Workbench version 20.0.4 (Qiagen, Hilden, Germany) was used for data analysis. Quality control of the raw reads, including removal of adapter sequence and low-quality reads, was performed using the default settings of CLC Workbench. Consensus sequences were extracted from the mapped reads after simultaneous mapping of the reads to the following reference sequences: GenBank accession number NC_006307 for PB2, NC_006308 for PB1, NC_006309 for P3, NC_006310 for HE, NC_006311 for NP, NC_006312 for M, and NC_006306 for NS.

**FIG 1:**
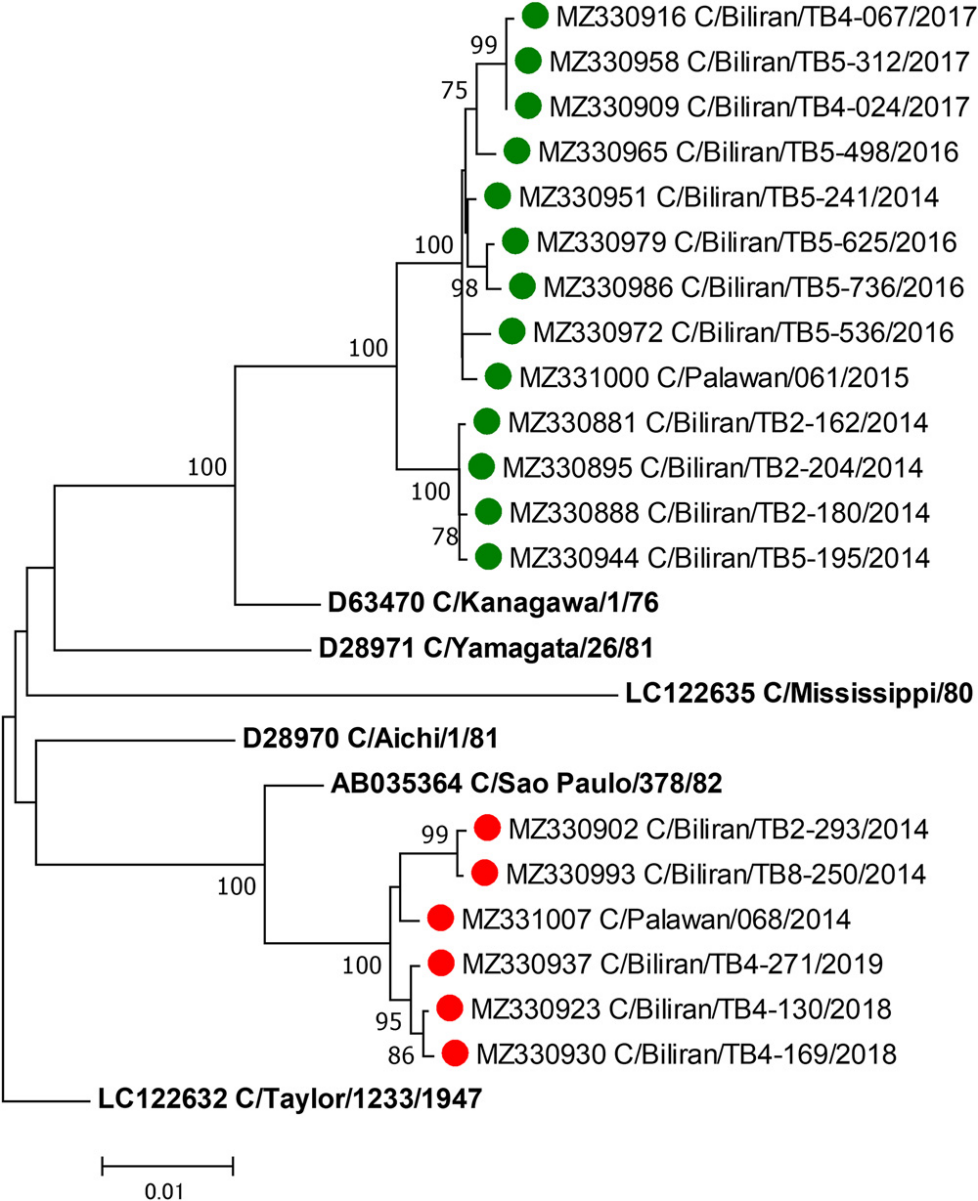
Phylogenetic tree of the influenza C virus hemagglutinin-esterase (HE) gene. For this analysis, the length of the coding region of the HE gene was 1,926 nucleotides (positions 64 to 1989). The phylogenetic tree was constructed using the neighbor-joining method in MEGA7 software, using T92 as the substitution model with gamma-distributed site heterogeneity. The bootstrap values were calculated from 1,000 replicates, and values greater than 75% are shown at the branches of the tree. Strains analyzed in this study are marked with circles: green circles for lineage C/Kanagawa and red circles for lineage C/Sao Paulo.

The average coverage per sample ranged from 5,390 to 152,413 reads (Table 1). The minimum and maximum depths of coverage in the CDS of all samples were 143 and 551,997 reads, respectively. The full length of the CDS was covered at more than 100× depth. Based on a phylogenetic analysis of the HE gene, the samples were divided into two lineages: C/Kanagawa and C/Sao Paulo (Fig. 1).

**TABLE 1:** Information on complete genome sequences of influenza C virus^*a*^

Strain	Yr	No. of reads	% GC	Avg coverage (×)	% of coding regions covered at ≥100×	Sequence Read Archive accession no.	GenBank accession no.	Gene length (bp)						
								PB2	PB1	P3	HE	NP	M	NS
C/Palawan/068/2014	2014	44,582,786	52.35	62,205	94.47	SRR14537525	MZ331007–MZ331013	2,325	2,265	2,130	1,968	1,698	1,125	862
C/Biliran/TB5-195/2014	2014	43,211,150	54.41	13,916	94.39	SRR14537524	MZ330944–MZ330950	2,325	2,265	2,130	1,968	1,698	1,125	862
C/Biliran/TB5-241/2014	2014	53,471,664	53.69	34,393	94.47	SRR14537514	MZ330951–MZ330957	2,325	2,265	2,130	1,968	1,698	1,125	862
C/Biliran/TB2-162/2014	2014	57,572,618	55.40	9,978	94.41	SRR14537513	MZ330881–MZ330887	2,325	2,265	2,130	1,968	1,698	1,125	862
C/Biliran/TB2-180/2014	2014	48,560,152	54.76	5,390	94.40	SRR14537512	MZ330888–MZ330894	2,325	2,265	2,130	1,968	1,698	1,125	862
C/Biliran/TB8-250/2014	2014	49,854,394	54.55	20,153	94.37	SRR14537511	MZ330993–MZ330999	2,325	2,265	2,130	1,968	1,698	1,125	862
C/Biliran/TB2-204/2014	2014	52,851,942	53.35	18,793	94.51	SRR14537510	MZ330895–MZ330901	2,325	2,265	2,130	1,968	1,698	1,125	862
C/Biliran/TB2-293/2014	2014	45,432,860	53.49	20,277	94.39	SRR14537509	MZ330902–MZ330908	2,325	2,265	2,130	1,968	1,698	1,125	862
C/Palawan/061/2015	2015	44,671,646	53.57	31,770	94.46	SRR14537508	MZ331000–MZ331006	2,325	2,265	2,130	1,968	1,698	1,125	862
C/Biliran/TB5-498/2016	2016	61,000,696	54.27	63,616	94.45	SRR14537507	MZ330965–MZ330971	2,325	2,265	2,130	1,968	1,698	1,125	862
C/Biliran/TB5-536/2016	2016	62,519,482	52.71	81,834	94.42	SRR14537523	MZ330972–MZ330978	2,325	2,265	2,130	1,968	1,698	1,125	862
C/Biliran/TB5-625/2016	2016	57,831,760	49.51	100,708	94.52	SRR14537522	MZ330979–MZ330985	2,325	2,265	2,130	1,968	1,698	1,125	862
C/Biliran/TB5-736/2016	2016	48,786,384	53.59	38,654	94.51	SRR14537521	MZ330986–MZ330992	2,325	2,265	2,130	1,968	1,698	1,125	862
C/Biliran/TB4-024/2017	2017	62,691,494	52.84	88,608	94.55	SRR14537520	MZ330909–MZ330915	2,325	2,265	2,130	1,968	1,698	1,125	862
C/Biliran/TB5-312/2017	2017	62,459,368	54.84	60,857	94.47	SRR14537519	MZ330958–MZ330964	2,325	2,265	2,130	1,968	1,698	1,125	862
C/Biliran/TB4-067/2017	2017	62,583,132	55.01	47,907	94.48	SRR14537518	MZ330916–MZ330922	2,325	2,265	2,130	1,968	1,698	1,125	862
C/Biliran/TB4-130/2018	2018	42,157,534	51.59	79,847	94.48	SRR14537517	MZ330923–MZ330929	2,325	2,265	2,130	1,968	1,698	1,125	862
C/Biliran/TB4-169/2018	2018	49,578,338	49.16	152,413	94.58	SRR14537516	MZ330930–MZ330936	2,325	2,265	2,130	1,968	1,698	1,125	862
C/Biliran/TB4-271/2019	2019	47,001,394	56.25	10,687	94.50	SRR14537515	MZ330937–MZ330943	2,325	2,265	2,130	1,968	1,698	1,125	862

a The average coverage and percentage of coding regions covered at ≥100× were calculated for the complete sequence of the coding regions as follows: nucleotides 22 to 2346 for polymerase basic 2 (PB2), nucleotides 18 to 2282 for polymerase basic 1 (PB1), nucleotides 22 to 2151 for polymerase 3 (P3), nucleotides 22 to 1989 for hemagglutinin-esterase (HE), nucleotides 30 to 1727 for nucleoprotein (NP), nucleotides 26 to 1150 for matrix (M), and nucleotides 27 to 888 for the nonstructural (NS) gene.

These nearly complete genome sequences reveal the ICV lineages that circulated between 2014 and 2019 in the Philippines.

### Data availability.

The nearly complete genome sequences of 19 ICV isolates have been deposited in GenBank (accession numbers MZ330881 to MZ331013). The raw sequencing reads were deposited in the NCBI Sequence Read Archive under BioProject accession number PRJNA719209.
